# A Berlin Physician on the Treatment of Consumption

**Published:** 1888-11-10

**Authors:** 


### Within the Wards.

### A BERLIN PHYSICIAN ON THE TREATMENT OF CONSUMPTION.

All the world is interested in the treatment of consumption, and patients and their friends, who have an intelligent understanding of a few general principles, may often aid the physician very considerably in his management of cases. One of the doctors at the Lengerich Hospital, Berlin, has recently made certain suggestions which, though perfectly familiar to many medical men in this country, are worthy of careful consideration by those who suffer from chest disorder, or who have charge as doctors or nurses of phthisical patients. The origin of Dr. Halter's paper seems to have been the fact that lime-burners seldom suffer from pulmonary consumption. Is it really a fact that those who work about lime-kilns are free from phthisis? It is stated that in a certain country district, where consumption was extensively prevalent, no case of the disease had occurred among the men at the lime-kilns for fifteen years. This is, however, a limited experience. It is not sufficient of itself to found a dogmatic generalisation upon, but it is sufficient to suggest careful inquiry, and to induce all medical men in lime-burning districts to go back upon the history of men and families engaged in this particular industry, and also to commence a series of observations and records of those now at work. What does Dr. Halter consider to be the curative or rather the preventive element about lime-kilns? It is neither the dry soil of the limestone district nor the fine chalk dust inhaled by the workers. The essential thing, he considers to be the *hot dry air* which they breathe. The temperature in which they work is from 122 deg. F. to 156 deg. F. The relative moisture of the air never exceeds about 50 per cent. So far as regards dryness, therefore, the atmosphere is similar to that of such famous health resorts as Davos, Denver, San Remo, and Cairo. There is a physiological explanation which appeals to those who have studied the conditions most favourable to the decay of organic structures. It is known that animal and vegetable organisms when life is extinct tend to decay with more or less rapidity. Among the conditions most conducive to slow decay is great dryness of the atmosphere. When the dryness is accompanied by great heat the air is still more unfavourable for the development of those low organisms which accompany decay. The *Bacillus Tuberculosis*, for example, dies at a temperature of 106 deg. F. It is easy to see, therefore, how the lime burner, working in an atmosphere of 130 deg. F. should escape phthisis. It must be remembered, however, that all this proceeds upon the assumption that consumption depends exclusively upon a certain bacillus, and that to cure the disease all that is necessary is to destroy the bacillus. But as a matter of fact that view of the case has not yet got beyond the stage of plausible hypothesis. As a contribution towards the intelligent management of cases of consumption, Dr. Halter's paper is not without value.

